# A Study of Physico-Mechanical Properties of Hollow Glass Bubble, Jute Fibre and Rubber Powder Reinforced Polypropylene Compounds with and without MuCell^®^ Technology for Lightweight Applications

**DOI:** 10.3390/polym12112664

**Published:** 2020-11-12

**Authors:** Yinping Tao, Srichand Hinduja, Robert Heinemann, Anselmo Gomes, Paulo Jorge Bártolo

**Affiliations:** 1 Department of Mechanical, Aerospace and Civil Engineering, The University of Manchester, Manchester M13 9PL, UK; Yinping.tao@manchester.ac.uk (Y.T.); Robert.Heinemann@manchester.ac.uk (R.H.); 2 SET Europe Ltd., 15/17 Seddon Place, Stanley Industrial Estate, Skelmersdale, Lancashire WN8 8EB, UK; anselmo.gomes@sanko.co.uk

**Keywords:** lightweighting, injection moulding, MuCell^®^ foaming, filler hybridisation, composites

## Abstract

Lightweighting is one of the key solutions to reduce the carbon footprint of vehicles. Nowadays, it is still challenging to achieve this target because there is a conflict between the cost and final material performance, as well as the fact that many lightweight solutions are restricted to laboratory or small-scale production. In this work, a commercially feasible strategy was adopted to fabricate materials for lightweight applications. Hollow glass bubbles, jute fibres, and rubber powder were used as fillers with polypropylene as the base polymer. Various samples were fabricated using conventional and MuCell^®^ injection moulding. Their performance was then characterised by their density and morphological, mechanical, and rheological properties. A comparison among hybrid fillers/polypropylene compounds with and without MuCell^®^ technology was investigated. The filler hybridisation resulted in not only a density reduction of up to approximately 10%, but also improved tensile/flexural modulus and strength. The use of MuCell^®^ led to a further reduction in density of roughly 10%. Meanwhile, although some compounds fabricated by MuCell^®^ exhibited some deterioration in their tensile yield strength, tensile modulus, and impact strength, they maintained acceptable mechanical properties for automotive applications.

## 1. Introduction

Vehicle lightweighting has become one of the essential tasks in order to reduce fuel consumption, and thus to reduce CO_2_ emissions in view of EU legislation establishing a lower CO_2_ emission target for vehicles manufactured after 2020 [1,2]. In the automotive industry, injection moulding is a widely employed technology for mass production of car components due to its short cycle time. However, the main limitations of conventional injection moulding are the demanding clamping pressure and the relatively heavy weight exhibited by the resultant plastic components. In order to address this issue, thermoplastic manufacturing processes such as microcellular injection moulding (MuCell^®^) [3] have been developed to produce microcellular foam structures [4]. MuCell^®^ not only has the advantages of reduced material usage/cost and shorter cycle times, but also allows for more tool design freedom due to reduced polymer melt viscosity and clamping pressure [5]. The end product, microcellular foam, is lighter and possesses better thermal insulation when compared to components manufactured by conventional injection moulding [6,7].

Apart from the manufacturing process, material formulation also plays a strategic role in the weight reduction of plastic components. Polypropylene is one of the most commonly used polymers for automotive components. Fillers such as talc [8,9] and glass fibres [10,11] are often compounded with virgin polymers for reinforcement and economic considerations at a typical loading of 10–25 wt%. Although these fillers increase the stiffness, they have a relatively high density of 2.5–2.7 g/cm^3^, leading to an increase in the density of the resultant compounded polymer. Therefore, lightweight fillers are preferred. Recently, high-stiffness hollow glass bubbles (HGBs) have emerged as a potential substitute filler with a density of 0.21–0.68 g/cm^3^ [12] and have proved to be effective in weight reduction for thermoplastics [13]. For instance, Lee et al. [14] found that the incorporation of HGBs has a synergistic effect on improving the mechanical properties and decreasing the density in sheet moulding of a fibre-reinforced composite. Rupam et al. [15] observed a consistent density reduction with increasing HGB content and an improvement in tensile strength in a polypropylene composite containing up to 5 wt% of HGBs. They also reported a continuous increase in hardness but observed a reduction in impact strength. Furthermore, compared to silica-based glass fibres, plant-derived natural fibres, such as jute [16,17,18] and hemp [19], are attracting increasing attention as reinforcing fillers due to their biodegradability and sustainability. Moreover, their specific density is approximately half of that of glass fibres [20,21] and they are generally less brittle, allowing them to maintain their high aspect ratio during processing [10]. However, because of the incompatibility between polar natural fibres and non-polar polyolefin-type thermoplastics, there is poor adhesion at the polymer–fibre interface [22]. The use of commercially available coupling agents, such as maleic anhydride grafted polypropylene (MA-PP), can be an effective way to enhance interfacial bonding [23,24]. In addition, micro rubber powder is an effective filler to improve the impact strength of materials [25,26]. It has been shown experimentally that the incorporation of micro rubber powder into polymer compounds reduces the material’s brittleness [27].

In this paper, the synergistic filler hybridisation effect of HGBs and jute fibre on the density, mechanical properties (tensile, bending, and impact), and rheological properties of polypropylene-based compounds were investigated. Moreover, samples fabricated from conventional and MuCell^®^ injection moulding were compared, showing that MuCell^®^ leads to a further weight reduction with some deterioration in tensile yield strength, tensile modulus, and impact strength whilst the flexural strength and modulus are maintained.

## 2. Materials and Methods

### 2.1. Materials

Polypropylene (PP) copolymer (total polypropylene PPC6742) was used as the thermoplastic base polymer with a melt flow index (MFI) of 8 g/10 min. In the context of this paper, this polymer is referred to as PP Neat. Hollow glass bubbles (tradename: iM16k) with a density of 0.46 g/cm^3^ and an average diameter of 20 µm were purchased from 3M (Maplewood, MN, USA). Jute fibres (grade F501/400) were purchased from Schwarzwälder Textil-Werke Heinrich Kautzmann GmbH. (Schenkenzell, Germany) and 40′s Mesh SBR Rubber Crumbs were provided by SRC Products Ltd. (Stockport, UK) with an average particle size of 250 µm and a density of 0.73 g/cm^3^. Maleic anhydride grafted PP (Exxelor PO 1020; ExxonMobil Chemical (Europe), Machelen, Belgium) was used as a coupling agent to add polarity to polypropylene and to improve the bonding with fillers. Talc-reinforced PP, currently used for car components, was used to benchmark the properties of the new materials developed in this study. In the context of this paper, it is referred to as PP Current. Note that the base polymer of PP Current is not PPC6742.

### 2.2. Sample Preparation

Compounding was performed using a Coperion STS35 adv machine (Coperion, Stuttgart, Germany) with a length/diameter value of 40. The compounding temperature profiles over nine heating zones are listed in Table 1. The material formulations used are shown in Table 2. The rotational speed of the screw was 250 rpm. For the sake of simplicity and clarity, the various compounded materials are designated GB1, GB2, GB3, JUTE1, JUTE2, JUTE3, and PP Neat. These materials were then pelletised for subsequent use in injection moulding.

Conventional injection moulding was carried out on a Boy^®^ 22A Pro machine (Dr. Boy GmbH & Co. KG, Neustadt, Germany). The injection speed was 55 mm/s. MuCell^®^ injection moulding was performed on a 300-ton Engel Victory machine (ENGEL Austria GmbH, Schwertberg, Austria) equipped with MuCell^®^ technology (Trexel, Inc., Woburn, MA, USA). This machine had a screw diameter of 60 mm. The injection speed was 250 mm/s and the supercritical fluid nitrogen (SCF-N_2_) content was 0.7 wt%. This content is recommended in [28], where a maximum cell density was obtained at an SCF-N_2_ level of 0.7 wt% for a carbon fibre/polypropylene composite foam.

### 2.3. Characterisation

#### 2.3.1. Morphology and Filler Dispersion

Scanning electron microscopy was carried out using a FEI Quanta 200 (Thermo Fisher Scientific Inc., Waltham, MA, USA). The samples were immersed in liquid nitrogen for five minutes prior to fracture, and then were coated with 6 nm Au/Pt (40 wt%/60 wt%) using a Quorum Q150T ES sputter coater (Quorum, Laughton, UK) before imaging. Both secondary electron (SE) and backscattered electron (BSE) signals were used to determine surface morphology and filler phase distribution [29].

#### 2.3.2. Density Measurement

Density measurements were carried out using a Weightron Bilanciai Sartorius machine (Weightron Bilanciai Ltd, Chesterfield, UK), which utilises Archimedes’ principle. Plastic pellets were weighed both in air (*m_air_*) and water (*m_water_*) and the density of the test sample (*ρ_sample_*) was calculated as follows [6]:

(1) $$\rho_{sample}=\rho_{water}\frac{m_{air}}{m_{air}-m_{water}}$$

where *ρ_water_* is the density of water at 23 °C and atmospheric pressure.

#### 2.3.3. Mechanical Tests

Tensile tests were performed on an Instron 5969L2078 (Instron, High Wycombe, UK) using a 1 kN loadcell according to ISO 527. The test speed was 50 mm/min. A clip-on extensometer MTS with a gauge length of 25 mm was employed to record the yield strain of the test sample. The breaking strain was determined by the displacement of the frame minus the machine compliance.

Three-point bending tests were carried out on an Instron 3344 (Instron, High Wycombe, UK) using a 500 N loadcell according to ISO 178. The test samples were of size 80 × 10 × 4 mm^3^, with a span length of 64 mm. The tests were carried out at a speed of 5 mm/min.

Notched Izod impact tests were conducted on a Zwick Roell HIT Pendulum Impact Tester HIT 5.5P (Instron, High Wycombe, UK) according to ISO 180. For PP Current, HGB, and jute compound specimens, a 1 J hammer was used, whilst a 2.75 J hammer was employed for PP Neat specimens. The test samples were of size 80 × 10 × 4 mm^3^ with a 45° notch.

For each of the mechanical tests mentioned above, a minimum of five specimens per sample were used.

#### 2.3.4. Rheology

The rotational rheological analysis was performed on an AR-G2 rheometer (TA Instrument, New Castle, DE, USA). The lower plate was fixed on the machine, whilst the upper parallel plate was rotating. The distance between the plates was set as 1500 µm and the temperature as 200 °C (equivalent to the injection temperature). The test samples were 25 mm in diameter with a thickness of 1.54 mm and three tests were conducted for each material. The viscosity was recorded for increasing values of shear rate up to 50 s-^1^; beyond this value the results were not reliable due to the effect of the centrifugal force on the measuring system.

#### 2.3.5. Melt Flow Index (MFI) Measurement

Melt flow index tests, which are often used for controlling the fluidity of the melt [30], were carried out on a ZwickRoell Mflow machine (ZwickRoell, Leominster, UK) according to ISO 1133. A normal load of 2.16 kg was applied onto the molten polymer in a heated cylinder at a constant temperature of 230 °C.

## 3. Results

### 3.1. Interfacial Morphology and Filler Dispersion of Materials

**Figure 1:**
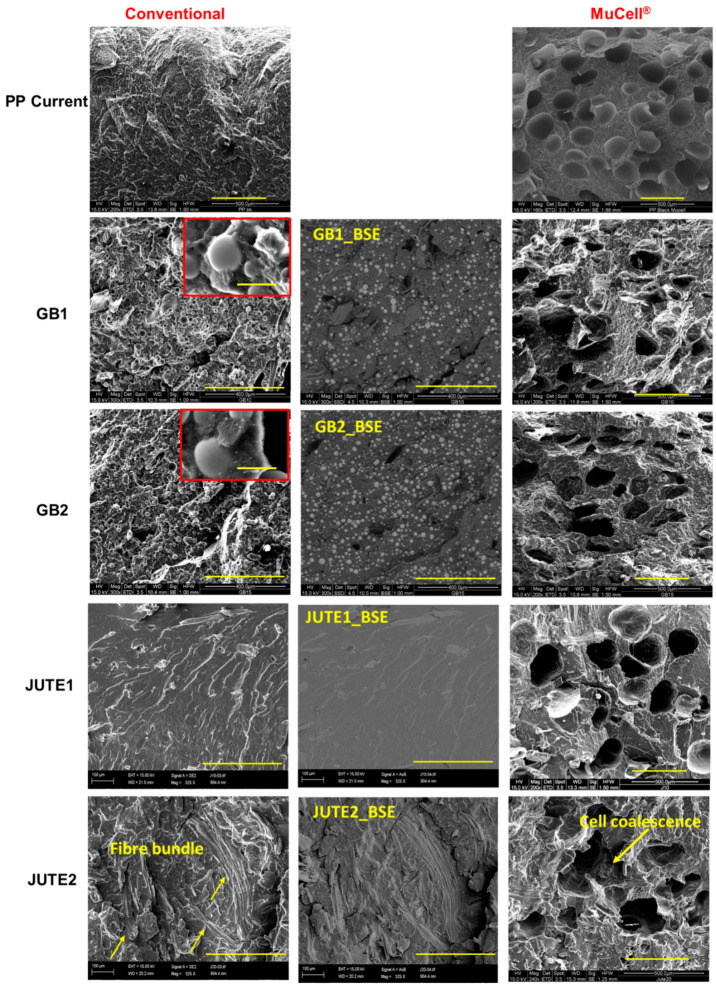
SEM images of the cryofracture surface morphology of GB1, GB2, JUTE1, JUTE2, and PP Current using conventional and MuCell^®^ injection moulding. Note: The scale in the individual images represents 400 µm, whilst the scale in the two inserted images represents 25 µm.

All of the materials listed in Table 2 were fabricated using both conventional and MuCell^®^ injection moulding. However, there were processing difficulties (short shot issues and occasional injection sprue blockage issues) when using MuCell^®^ to fabricate GB3 and JUTE3, which contained the largest percentage of hollow glass bubbles and jute fibres. Therefore, GB3 and JUTE3 do not have MuCell^®^ counterparts. The middle column in Figure 1 shows the filler phase dispersion within the matrix using the BSE signal, while those in the left and right columns reveal the surface morphology using the SE signal. It can be seen that the HGBs are evenly dispersed in the matrix without any visible agglomeration. The inserts for GB1 and GB2 show a good interface between the HGBs and polypropylene matrix. In the case of jute compounds, the fibres were well distributed in the matrix for JUTE1 whilst, in the case of JUTE2, some jute fibre bundles were observed. In terms of foamability in the MuCell^®^ process, PP Current has the best foamability among all of the compounds, indicated by more homogeneous cellular structures with an average pore diameter of 250 µm. In contrast, pores appear to be more elliptical in the compounded materials. Although previous research has shown that the presence of a small amount of fillers facilitates cell nucleation [13], the presence of high amounts of HGBs and jute fibres could interfere with cell growth, leading to cell collapse and coalescence. The reduced foamability is associated with their weak melt strength during foaming, where the cell walls are not able to retain the gas during the foaming/expansion process [31,32]. In addition, in the case of HGB compounds, the glass bubbles act as a lubricant, leading to easier sliding between polymer chains under extensional deformation and, hence, a lower melt strength [33].

### 3.2. Density

**Figure 2:**
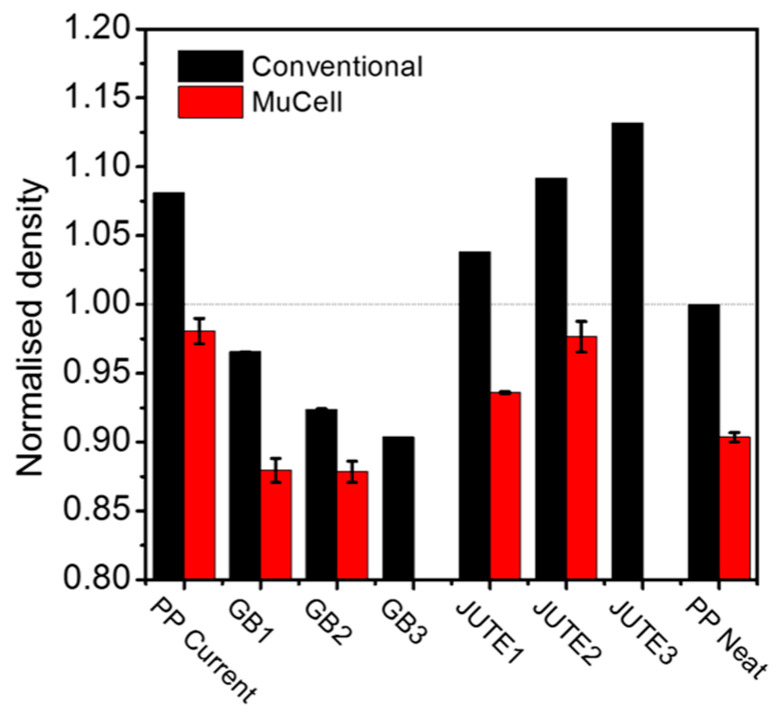
Normalised densities for PP Neat and different compounds using conventional and MuCell^®^ injection moulding. Note: The zero-length error bar in this figure is due to identical values of the density for each of the three specimens.

Figure 2 presents the normalised density values of various materials using conventional and MuCell^®^ injection moulding, and Table 3 summarises the normalised density changes as a result of a change in formulation and switching from conventional to MuCell^®^ injection moulding. Normalised density was calculated as the density of a compound divided by the density of PP Neat prepared by conventional injection moulding. The addition of jute fibres led to a slight increase in density. However, the increase was approximately 11% lower than that for materials reinforced with the same amount of glass fibres reported in the literature, which can be attributed to the difference in densities between jute and glass fibres [34]. Compared to PP Neat, the introduction of HGBs had a significant impact on the density, e.g., there was a 9.6% reduction in the case of GB3. Since GB3 also consists of a certain amount of jute for toughness and damage tolerance considerations, the reduction in density in response to compounding PP with only HGBs would be even greater.

Using MuCell^®^, the bulk density of the materials was further reduced by approximately 9–10% when compared to conventional injection moulding, with the exception of GB2, where the density decreased by only 5%. The level of reduction in bulk density was related to the porosity of the materials, which depends on their foamability. As discussed in Section 3.1, due to the large amount of fillers GB2 possesses (25wt% in total), these fillers interfere with cell growth, leading to cell collapse and coalescence. The presence of a large amount of HGBs acts as a lubricant, promoting the sliding of polymer chains and leading to reduced melt strength of GB2, resulting in reduced foamability and porosity.

### 3.3. Rheological Properties

**Figure 3:**
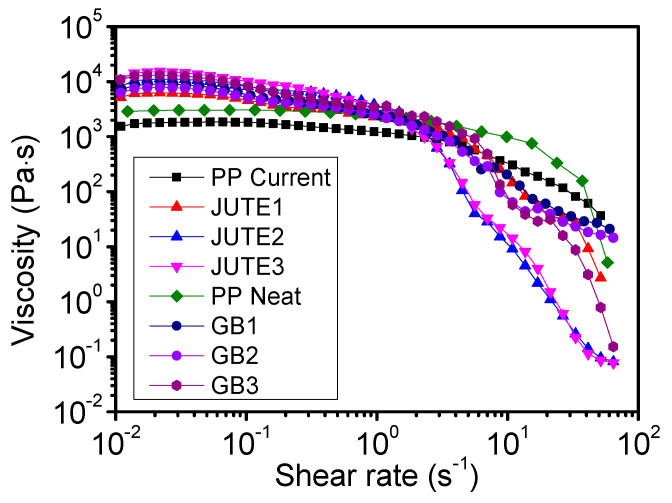
Effect of shear rate on shear viscosity at a temperature of 200 °C for different materials.

**Figure 4:**
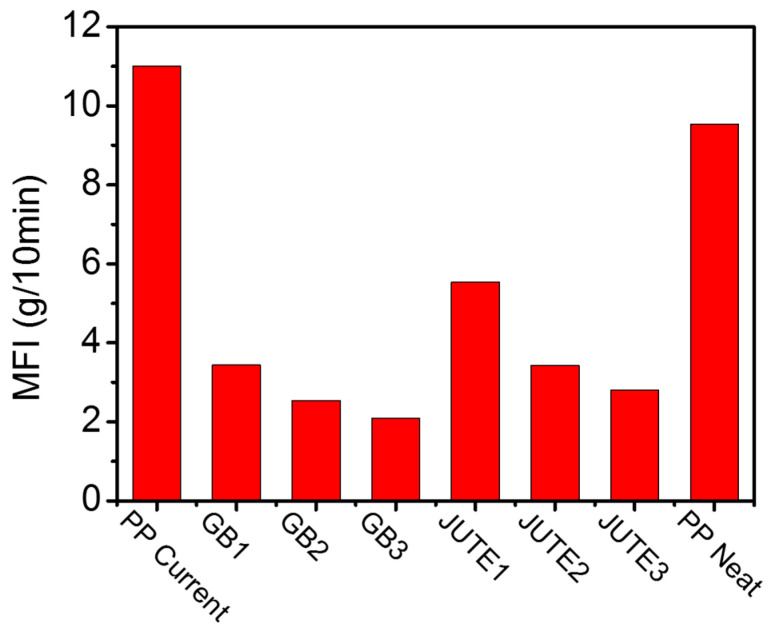
Melt flow index (MFI) values for different materials.

Figure 3 shows the effect of shear rate on the shear viscosity at a temperature of 200 °C for various compounds. It can be seen that a higher loading of fillers increases the viscosity of both jute and HGB compounds in the low shear rate regime, a trend that is similar to that expressed by the Maron–Pierce relationship [35]. This is also reflected in the MFI values of the materials, as shown in Figure 4. With increasing shear rate up to 50 s^−^^1^, the viscosity of filled polymers drops dramatically as the mechanism of polymer chain orientation and disentanglement prevails at a higher shear rate [36]. Moreover, highly filled compounds (JUTE2 and JUTE3) experience an earlier drop in viscosity, probably due to the breakdown of networks formed by the high aspect ratio of fibrous fillers [37].

### 3.4. Mechanical Properties

**Figure 5:**
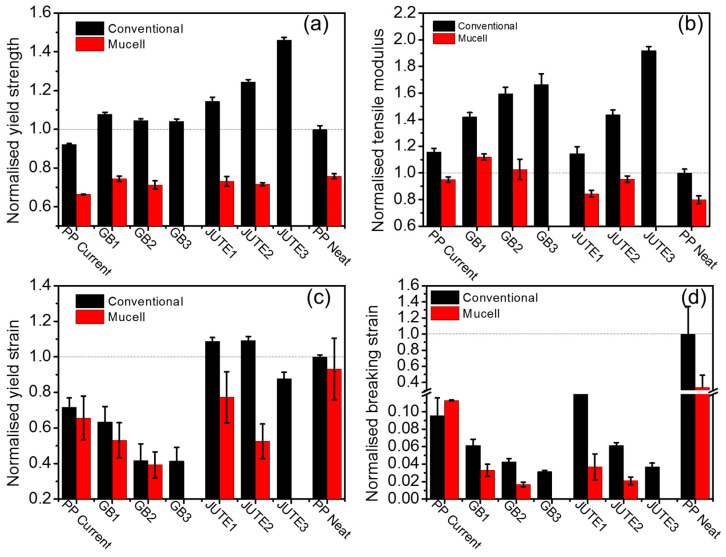
Normalised tensile properties of different materials: (**a**) yield strength; (**b**) tensile modulus; (**c**) yield strain; (**d**) breaking strain. Note: The data presented were normalised with respect to the corresponding data obtained for conventional injected PP Neat, indicated by the dashed line.

**Figure 6:**
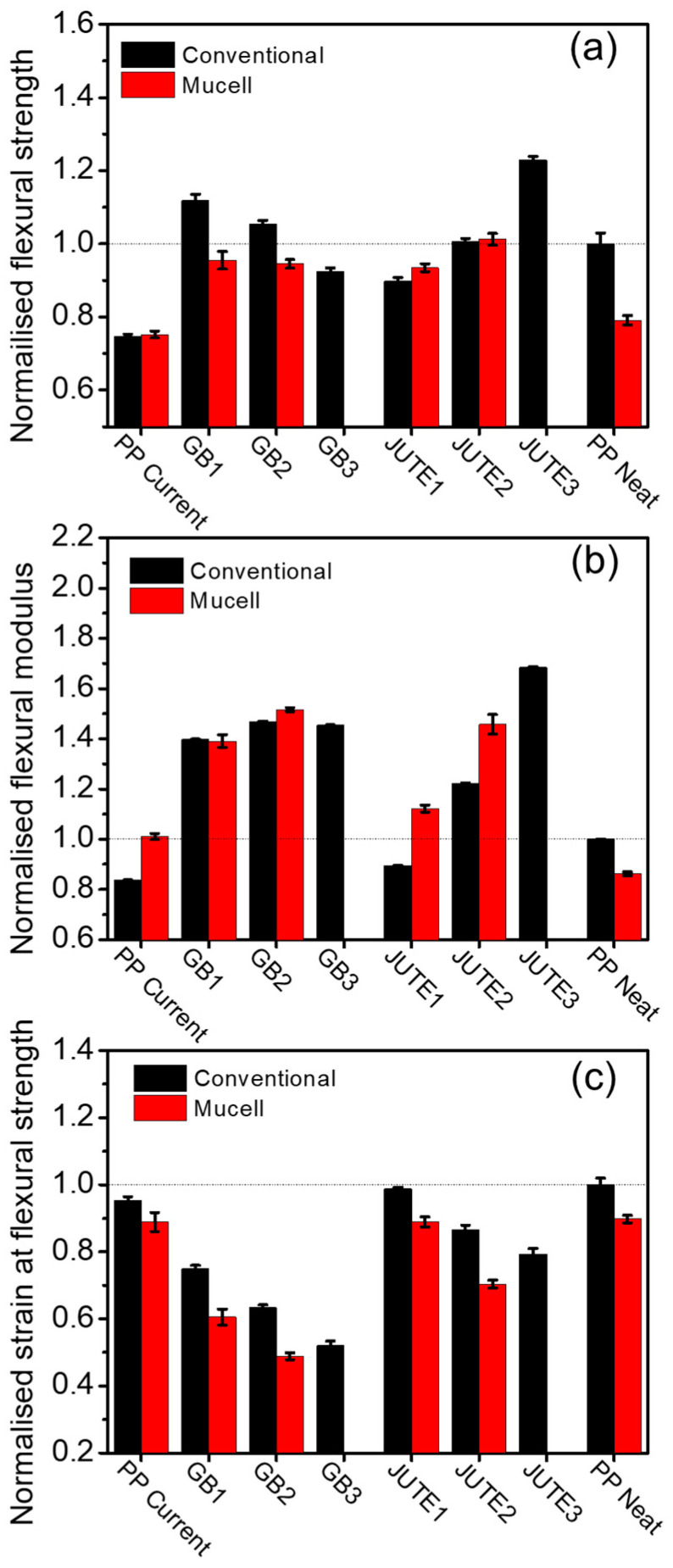
Normalised flexural properties: (**a**) flexural strength; (**b**) flexural modulus; (**c**) strain at flexural strength. Note: The data presented were normalised with respect to the corresponding data obtained for conventional injected PP Neat, indicated by the dashed line. Normalised breaking strain information was excluded as PP Neat did not fracture during the experiment.

**Figure 7:**
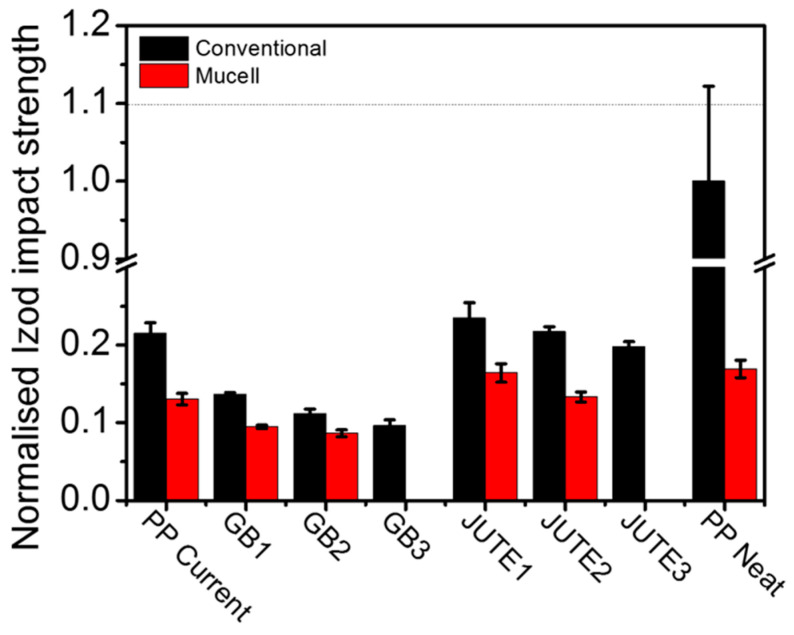
Normalised Izod impact strength of different materials. Note: The data presented were normalised with respect to the corresponding data obtained for conventional injected PP Neat, indicated by the dashed line.

To understand the reinforcing effect of hybrid fillers, the tensile, flexural, and impact properties were evaluated, and the results are summarised in Figure 5, Figure 6 and Figure 7. All of the data were normalised with respect to the corresponding data obtained from conventional moulded PP Neat, which is shown in Table S1 (see Supplementary Materials). When using conventional injection moulding, comparing the jute compounds with PP Neat, Figure 5a,b show that both the tensile yield strength and tensile modulus increase with an increasing amount of jute fibres, thus confirming the reinforcing effect of jute. This can be explained by the fact that the external load was effectively transferred from the PP matrix to the jute fibres [38]. Comparing JUTE1 with GB1, there is a slight reduction in the yield strength with the addition of HGB. Among the HGB compounds, the tensile modulus increases with an increase in HGB content whilst the tensile yield strength experiences a slight decrease. Nevertheless, the yield strength of the HGB compounds is still greater than that of PP Neat, as shown in Figure 5a,b. The incorporation of glass bubbles lowers the yield strength (weakening effect), but it improves the modulus of the material (stiffening effect). By making use of the hybridisation effect of jute and HGB fillers, a relatively strong material can be obtained without compromising the material’s density. At the same time, the yield strain and breaking strain are gradually reduced with increasing filler contents for both jute and HGB compounds, see Figure 5c,d. This can be attributed to the reduced polymer chain mobility in the presence of fillers [39].

The MuCell^®^ moulded materials experienced a reduction in their tensile strength (25–42%), tensile modulus (20–56%), yield strain (2–10%) and breaking strain (2–9%) when compared to their conventional counterparts. This reduction is due to the non-uniform porous structure of the materials, as revealed by the SEM images (Figure 1), especially in highly filled polymer compounds. Because of the existence of fillers and the weak melt strength of materials, the resulting porous structures are less uniform. In spite of these reductions, it is pertinent to note that a lower value in certain properties does not render it unsuitable. These reductions are acceptable for an application as long as its strength and modulus properties are comparable with those of the MuCell^®^ counterpart of PP Current, the benchmark material.

The flexural properties for conventional moulded samples (black column) show a similar trend in the tensile properties, that is, a reduction in strain at flexural strength and an increase in modulus with increasing filler loading (Figure 6). The normalised breaking strain information is not shown as PP Neat did not fracture during the experiment, making normalisation of this series of data impossible. In contrast, the flexural strength and modulus of the MuCell^®^ samples did not see a significant deterioration in spite of the porosity introduced in the materials, and in certain cases (for example, in the case of the flexural modulus of GB2), there was even a slight improvement. This is due to the fact that the bending strength and modulus are strongly influenced by the compact skin layer (the thickness of the skin layer is around 0.4 mm) where the maximum stress occurs [40]. Fillers are pushed towards the skin layer in the case of MuCell^®^ samples during foaming.

Figure 7 shows the normalised impact strength of various materials. Although previous research shows that adding short fibres into a matrix material tends to improve its toughness [20], this research indicates that, when comparing jute compounds with PP Neat using conventional injection moulding, the addition of jute fibres reduces the impact strength. This may be caused by the presence of fibre bundles leading to premature failure. Research has shown that micro rubber powders tend to improve the toughness of materials [25,26], but in this study, it was observed that the addition of micro rubber powders did not counteract the detrimental effect caused by the jute fibres. This effect might be compensated by incorporating a sufficient amount of rubber powder in a future study. The incorporation of jute had a significant negative effect on the impact strength of the compound, which was worsened by an increase in the amount of jute. The impact strength was further worsened by adding HGBs, and the extent of this reduction was proportional to the amount of HGBs added to the compound. Compared to conventional moulding, the MuCell^®^ counterparts exhibited a lower impact strength, reduced by 4–8%, which can be attributed to the presence of a non-uniform cell structure, as shown in Figure 1.

## 4. Conclusions

In this study, both hybrid fillers and MuCell^®^ technology were utilised for the fabrication of lightweight materials. A comparison between materials fabricated using conventional and MuCell^®^ injection moulding was made, and the following conclusions can be drawn:

- The combination of filler hybridisation and MuCell^®^ technology can lead to a total weight reduction of up to 20% when compared to the current plastic materials available on the market. For instance, replacing PP Current with GB3 using conventional moulding or with GB2 using MuCell^®^ moulding can lead to a weight reduction of 18.7% and 16.4%, respectively.
- Owing to the incorporation of high-stiffness glass bubbles and reinforcing jute fibres, the newly developed PP compounds manufactured by conventional moulding exhibited increased tensile and flexural modulus and strength compared to PP Current, which is currently used for automotive components.
- Materials fabricated by MuCell^®^ exhibited some deterioration in their tensile modulus, tensile breaking strain, and impact strength, but they still possess acceptable mechanical properties for automotive applications.
- Investigating the influence of shear rate on the viscosity of compounded materials revealed that highly filled compounds (JUTE2 and JUTE3) experience an earlier drop in viscosity with increasing shear rate, probably due to the breakdown of the networks formed by the high aspect ratio fibrous fillers.

As a result of the combination of reduced weight and acceptable mechanical properties, the newly developed materials are promising candidates for the large-scale industrial manufacturing of lightweight components.

## Figures and Tables

**Table 1:** Temperature profiles over nine heating zones for hollow glass bubbles (HGBs) and jute compounds.

	Temperature (°C)								
Materials	Zone1	Zone2	Zone3	Zone4	Zone5	Zone6	Zone7	Zone8	Zone9
HGB compounds	190	190	190	190	200	200	200	200	200
Jute compounds	180	185	190	190	190	190	190	190	200

**Table 2:** Material formulations used in this study.

Designation	PPC6742 (wt%)	HGB (wt%)	Jute (wt%)	Rubber Powder (wt%)	MA-PP (wt%)
GB1	78%	10%	10%	-	2%
GB2	73%	15%	10%	-	2%
GB3	68%	20%	10%	-	2%
JUTE1	88%	-	10%	-	2%
JUTE2	74%	-	20%	4%	2%
JUTE3	64%	-	30%	4%	2%

**Table 3:** Normalised density changes as a result of a change in formulation and switching from conventional to MuCell^®^ injection moulding.

Sample Name	Density Change Caused by Formulation (%)	Bulk Density Change Caused by Process (%)
PP Neat	0	−9.6
GB1	−3.5	−8.9
GB2	−7.6	−4.9
GB3	−9.6	N/A
JUTE1	+3.8	−9.9
JUTE2	+9.2	−10.6
JUTE3	+13.2	N/A
PP Current	N/A	−9.3

Note: (−) and (+) denote a reduction and increase in density values, respectively; bulk density loss = (density using conventional process—density using MuCell^®^ process)/density using conventional process × 100%.

## Supplementary Materials

The following are available online at https://www.mdpi.com/2073-4360/12/11/2664/s1, Table S1: The mechanical properties of PP Neat using conventional injection moulding.
