# Supplementary material for: A Study of Physico-Mechanical Properties of Hollow Glass Bubble, Jute Fibre and Rubber Powder Reinforced Polypropylene Compounds with and without MuCell® Technology for Lightweight Applications
Source: Polymers (Basel). 2020 Nov 12;12(11):2664. doi: 10.3390/polym12112664 (PMC7697613; doi:10.3390/polym12112664)
Supplement: Supplementary file 1 [file polymers-12-02664-s001.pdf]

Supplementary Materials for

Article

A study of physico-mechanical properties of hollow glass bubble, jute fibre and rubber powder reinforced polypropylene compounds with and without MuCell technology for lightweight applications

Yinping Tao[1], Srichand Hinduja[1], Robert Heinemann[1], Anselmo Gomes[2], Paulo Jorge Bártolo[1]

1. Department of Mechanical, Aerospace and Civil Engineering, The University of Manchester, Manchester, M13 9PL, UK

2. SET Europe Ltd, 15/17 Seddon Place, Stanley Industrial Estate, Skelmersdale, Lancashire, WN8 8EB, UK

E-mail: sri.hinduja@manchester.ac.uk; paulojorge.dasilvabartolo@manchester.ac.uk

Table S1. The mechanical properties of PP Neat using conventional injection moulding.

Property	Unit	PP Neat
Tensile yield strength	MPa	23.61±0.43
Tensile yield strain	%	5.36±0.06
Tensile breaking strain	%	157.12±53.64
Tensile modulus	MPa	1578.00±45.46
Flexural strength	MPa	36.22±1.07
Flexural modulus	MPa	1320.6±36.24
Flexural strain at flexural strength	%	6.77±0.13
Izod impact strength	KJ/m2	37.11±4.54
